# Enhanced Degradation of Phenol in Aqueous Solution via Persulfate Activation by Sulfur-Doped Biochar: Insights into Catalytic Mechanisms and Structural Properties

**DOI:** 10.3390/nano15130979

**Published:** 2025-06-24

**Authors:** Guanyu Wang, Lihong Kou, Chenghao Li, Bing Xu, Yuanfeng Wu

**Affiliations:** 1 China Coal Science and Technology Research Institute Co., Ltd., Beijing 100013, China; siyueming@126.com (G.W.); klhklhklh007@126.com (L.K.); 2 Henan Key Laboratory of Coal Green Conversion, College of Chemistry and Chemical Engineering, Henan Polytechnic University, Jiaozuo 454003, China; lichenghaohpu@163.com

**Keywords:** sulfur-doped biochar, persulfate, phenol degradation, density functional theory

## Abstract

In this study, sulfur-doped biochar (SBC) was successfully synthesized using peanut shells as the raw material and sulfur powder as the sulfur source. The composition, structural characteristics, and catalytic performance of SBC in the degradation of phenol via persulfate (PDS) activation were systematically investigated. Characterization results demonstrate that the prepared SBC exhibited a typical lamellar structure with abundant pores and fissures on its surface. XPS analysis confirmed the successful incorporation of sulfur into the biochar matrix, primarily in the form of thiophene. Under the optimized condition of a 20% sulfur doping ratio, the SBC exhibited high efficiency in activating PDS, achieving a phenol degradation rate of 97%. Remarkably, the removal rate remained at 81% even after the fifth cycle, indicating excellent cyclic stability. Density functional theory (DFT) calculations and electrochemical impedance spectroscopy (EIS) measurements further revealed that sulfur doping significantly modified the electron density distribution of the biochar, reducing its surface electrochemical impedance from 32.88 Ω to 13.64 Ω. This reduction facilitated efficient electron transfer during the catalytic process. This study provides both experimental and theoretical insights into the charge distribution characteristics of sulfur-doped biochar, offering valuable references for understanding the mechanism of PDS activation by SBC.

## 1. Introduction

Phenolic compounds, as representative organic pollutants widely used in industry, are difficult to degrade due to their stable chemical structures, exhibiting significant biological toxicity and environmental hazards [1,2,3,4,5]. Therefore, the development of efficient, economical, and environmentally friendly technologies for treating phenolic wastewater is of great importance [6]. Currently, technologies such as adsorption [7,8], biodegradation [9,10,11], and photo/electrocatalysis [12,13,14,15] are widely applied in the treatment of phenolic wastewater. However, these technologies still face numerous challenges in practical applications, including difficulties in adsorbent recovery [16], the significant influence of temperature on microbial communities [17], and low utilization rates of light/electric energy [18]. In recent years, advanced oxidation processes based on persulfate have been recognized as one of the effective methods for treating phenolic wastewater due to their excellent oxidation efficiency, economic feasibility, and environmental friendliness [19,20,21,22,23].

Studies have shown that carbonaceous materials (such as reduced graphene oxide, activated carbon, biochar, carbon nanotubes, etc.) can effectively activate persulfate to degrade phenolic pollutants [24,25]. For example, Yang et al. significantly improved the removal rate of phenolic pollutants through acid-modified carbon nanotubes and identified edge defect structures and sp^2^ hybridized carbon as key factors enhancing persulfate activation [26]. However, materials such as carbon nanotubes, graphene, and carbon quantum dots are costly, limiting their practical applications. In contrast, biochar, an environmentally friendly carbonaceous material prepared under oxygen-limited conditions, has been proven to exhibit excellent performance in persulfate activation [27,28].

To further enhance the catalytic performance of biochar, element doping technology has been widely applied in the synthesis of biochar [29,30,31]. For instance, Yang et al. loaded cobalt onto biochar through a pyrolysis process to synthesize Co-BC and studied its performance in activating persulfate to degrade amaranth red and acetaminophen, achieving a degradation efficiency of up to 96% [32]. However, metal-doped biochar faces the issue of metal ion leaching, which may lead to secondary pollution [33].

Sulfur doping, as a simple and effective method, can significantly improve the catalytic ability of biochar [34]. Research indicates that sulfur atoms can alter interfacial reactions, form more orbital overlaps, and accelerate electron transfer due to differences in electronegativity. Additionally, the decomposition products of sulfur sources at high temperatures can significantly improve the pore structure of carbonaceous materials, thereby enhancing the catalytic activity of biochar [35,36].

In this study, sulfur-doped biochar was successfully synthesized using peanut shells and sulfur powder as raw materials. The physicochemical properties of the biochar were systematically characterized using techniques such as SEM, BET, Raman, and XPS. On this basis, the catalytic degradation performance of sulfur-doped biochar for phenolic pollutants in a persulfate system under different preparation parameters and reaction conditions was investigated, revealing the structure–activity relationship between the biochar’s structure and its degradation efficiency. Additionally, by combining DFT calculations and EIS experiments, the effects of sulfur doping on the charge density distribution and resistance of the biochar were analyzed, and a degradation mechanism for phenolic pollutants was proposed. The innovations of this study include the following: (1) For the first time, peanut shells were used as raw materials to develop highly efficient persulfate-activating biochar through sulfur doping, providing a new approach for the development of low-cost, high-performance catalysts. (2) Through DFT and EIS experiments, the enhancement mechanism of sulfur doping on catalytic performance was elucidated at the electronic structure level, offering theoretical guidance for catalyst design. (3) The relationship between the pore structure, surface properties, and catalytic performance of sulfur-doped biochar was systematically studied, providing experimental evidence for optimizing catalyst performance. This study not only offers a new method for the efficient treatment of phenolic wastewater, but also lays a theoretical foundation for the development and application of biochar-based catalysts.

## 2. Materials and Methods

The experimental materials and instruments, the preparation procedures for biochar, and the analytical techniques employed are detailed in the Supplementary Materials.

## 3. Results and Discussion

### 3.1. Material Characterizations

**Figure 1:**
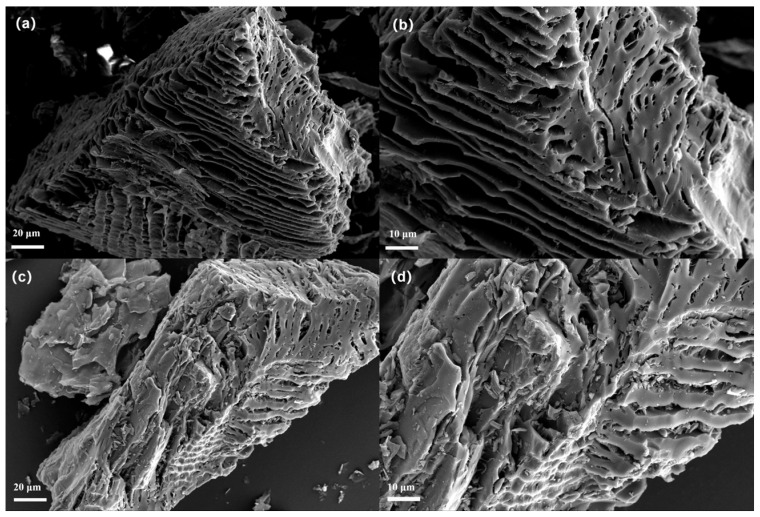
SEM images: (**a**,**b**) BC-800; (**c**,**d**) SBC-700.

The SEM images of the as-prepared BC-800 are presented in Figure 1a,b, whereas Figure 1c,d displays the images of SBC-700. From the images, it is evident that BC-800 exhibits a layered structure with uniformly distributed pores across its layers. Furthermore, in an effort to preserve the abundant pore structure of BC-800, the modified biochar demonstrated an even richer pore network, characterized by an increased number of cracks. This enhanced porosity provides additional channels for pollutant adsorption and the activation of PDS.

**Figure 2:**
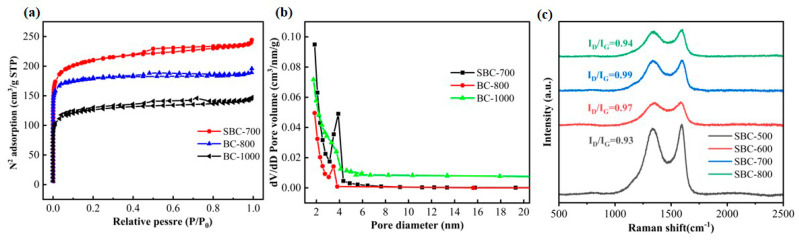
Nitrogen adsorption–desorption isotherms (**a**), pore size distribution curves (**b**), and Raman spectra (**c**).

Figure 2 presents the nitrogen adsorption–desorption isotherms and pore size distribution diagrams of different sulfur-doped biochars. The analysis results indicate that the nitrogen adsorption–desorption isotherms of the biochars conform to the Type I adsorption isotherm, with a pore size distribution ranging from 2 to 6 nm, predominantly characterized by a microporous structure. Additionally, Table 1 provides detailed microstructural parameters of the different sulfur-doped biochars, including a specific surface area. As shown in Table 1, the specific surface area of the obtained biochars gradually increased from 711 m^2^/g to 756 m^2^/g as the co-pyrolysis temperature rose from 500 °C to 700 °C. However, when the temperature further increased to 800 °C, the specific surface area decreased to 730 m^2^/g. This phenomenon can be attributed to the following reasons: lower co-pyrolysis temperatures are unfavorable for effective sulfur doping, while higher temperatures may lead to increased sulfur volatilization, thereby affecting the specific surface area. Notably, compared to BC-800, SBC-700 exhibits a higher specific surface area, indicating that SBC-700 possesses a stronger capability for the catalytic degradation of phenol.

Figure 2c presents the Raman spectroscopy analysis results of SBC. Based on previous studies, the following conclusions can be drawn: heteroatom doping can significantly enhance the defect degree of biochar [37,38]; as the carbonization temperature increases from 500 °C to 700 °C, the defect degree of SBC further increases. However, when the carbonization temperature continues to rise to 800 °C, the ID/IG ratio of SBC decreases. This indicates that, within a certain temperature range, both increasing the carbonization temperature and introducing heteroatom doping can effectively increase the defect degree of biochar [39], but the selection of the temperature range needs to be optimized. Additionally, changes in these defect structures may have significant impacts on the adsorption performance, electrochemical properties, and other characteristics of biochar [40].

**Figure 3:**
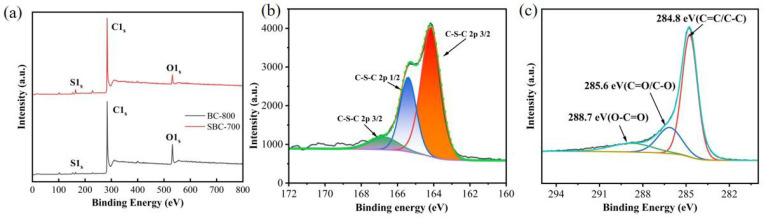
XPS spectra (**a**) and peak deconvolution analysis (**b**,**c**).

The surface chemical element composition and valence states of BC-800 and SBC-700 samples were characterized using XPS, and the results are shown in Figure 3. The high-resolution spectral peaks of S 2p appeared at 162.4, 164, 165.2, 166.1, and 170.03 eV, corresponding to CS2, C-S-S (S 2p3/2), C-S-S (S 2p1/2), C-SO-C, and CS2 (S 2p3/2), respectively [41,42,43]. Additionally, the characteristic peaks of C1s appeared at binding energies of 284.8, 285.6, and 288.7 eV, which are attributed to graphitic and aliphatic carbon (C sp2 and C sp3), ether bonds (C=O), and hydroxyl groups (C-O), respectively [44]. Notably, the C=O bond has been previously reported to effectively promote catalytic degradation reactions in PDS activation [45]. Table 2 provides information on the relative content of elements. As can be seen from Table 2, sulfur was successfully introduced into the biochar matrix, with its content increasing from 1.04% before doping to 3.15% after doping.

### 3.2. Catalytic Degradation of Phenol Using the Biochar

#### 3.2.1. Effect of Biochar Composition on Phenol’s Catalytic Degradation

**Figure 4:**
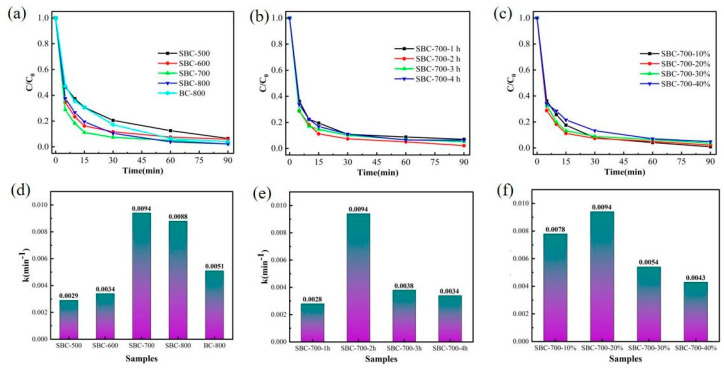
Effects of co-pyrolysis temperature (**a**), co-pyrolysis time (**b**), and doping ratios (**c**) on the degradation rate, along with the corresponding reaction rate constants derived from the pseudo-second-order kinetic fitting (**d**–**f**).

The results of phenol degradation by biochar are shown in Figure 4a. As can be seen from the figure, the sulfur-doped biochar exhibits superior catalytic degradation performance for phenol compared to the undoped biochar. As the co-pyrolysis temperature increased from 500 °C to 700 °C, the phenol removal rate of the prepared biochar gradually increased; however, when the co-pyrolysis temperature continued to rise to 800 °C, the removal rate decreased instead. The biochar prepared at 700 °C demonstrated the highest catalytic degradation rate and was able to degrade most of the phenol within 30 min. After doping, the difference in electronegative S atoms could regulate the charge distribution of surrounding carbon atoms, leading to an uneven distribution of carbon ring charge density, thereby breaking the catalytic inertia of the biochar and significantly accelerating phenol degradation [46,47]. Higher carbonization temperatures may not be conducive to the retention of S on the biochar, resulting in a reduction in catalytic sites, which significantly impacts the catalytic degradation of phenol. SBC-700 exhibited the highest phenol degradation rate and degradation efficiency, which is positively correlated with its largest specific surface area and highest defect density. These results indicate that co-pyrolysis temperature and S doping have a significant impact on the catalytic performance of biochar, and optimizing these parameters can significantly enhance its ability to degrade pollutants.

The effect of co-pyrolysis time on the catalytic degradation performance of phenol by the prepared biochar was investigated, and the results are shown in Figure 4b. After treating phenol-simulated wastewater with sulfur-doped biochar (SBC) for 90 min, it was observed that the catalytic degradation efficiency of phenol by four different SBCs tended to stabilize. As the pyrolysis time increased from 1 h to 2 h, the degradation rate of phenol significantly improved, indicating that appropriately extending the pyrolysis time helps enhance the catalytic activity of the biochar. However, when the pyrolysis time exceeded 2 h and continued to increase, the degradation efficiency gradually decreased, which might be attributed to the reduction in active sites on the biochar surface or structural changes caused by excessive pyrolysis time. Notably, SBCs prepared with pyrolysis times of 2 h and 3 h both exhibited high catalytic degradation rates, and their effects were essentially consistent, suggesting that within the range of 2 to 3 h, the pyrolysis time had a minor impact on catalytic performance. Based on the experimental results and analysis, the optimal pyrolysis time for sulfur-doped biochar was determined to be 2 h, ensuring high catalytic efficiency while optimizing the preparation process.

Figure 4c illustrates the performance changes in the degradation of phenol by persulfate activated with biochar at varying sulfur doping ratios. The experimental results indicate that as the sulfur doping ratio gradually increases from 10% to 40%, the catalytic degradation efficiency of phenol by biochar initially shows a significant improvement, followed by a gradual decline. Among the samples, SBC-700-20% with a sulfur doping ratio of 20% exhibits the most outstanding degradation performance, achieving a catalytic degradation rate of phenol as high as 89% within 15 min, which is significantly superior to samples with other doping ratios. This enhancement in efficiency is primarily attributed to the introduction of sulfur atoms altering the electronic structure of the biochar, leading to an uneven charge distribution and thereby forming more catalytic active sites on its surface, which enhances the activation capability of persulfate. However, when the sulfur doping ratio exceeds 20%, the excessive introduction of heteroatoms may disrupt the original structure of the biochar, resulting in reduced porosity or a decrease in active sites, thereby weakening the catalytic performance. Based on the experimental results and mechanistic analysis, the optimal sulfur doping ratio is determined to be 20%, ensuring efficient catalytic degradation of phenol while maintaining the structural stability of the biochar.

Figure 4d–f reveals the reaction rate constants for the degradation of phenol using activated persulfate (PDS) with different biochars. The findings demonstrate that the rate constant of SBC-700 is 1.75 times higher than that of pure biochar, indicating that sulfur doping significantly enhances the catalytic activity of biochar. This improvement can be attributed to the introduction of sulfur atoms, which modify the electronic properties of the biochar, creating more active sites and facilitating the activation of persulfate for efficient phenol degradation. The results highlight the critical role of sulfur doping in optimizing the catalytic performance of biochar in advanced oxidation processes.

#### 3.2.2. Effect of Varied Reaction Conditions in the PDS System on Phenol Degradation

**Figure 5:**
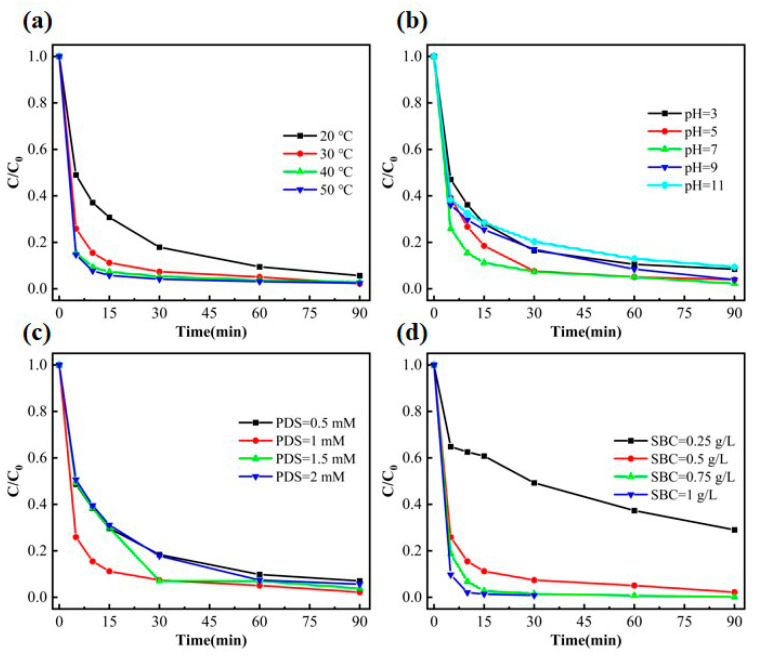
Effects of temperature (**a**), pH (**b**), PDS dosage (**c**), and SBC concentration (**d**) on phenol degradation efficiency.

To investigate the effect of reaction temperature on the catalytic degradation of phenol by SBC, the experiment systematically studied the catalytic degradation performance of SBC-700-20% on phenol under different temperature conditions (20 °C, 30 °C, 40 °C, and 50 °C), as shown in Figure 5a. The experimental results indicate that although the degradation rate of phenol is relatively low at a low temperature (20 °C), the degradation efficiency can still reach 87% after a certain reaction time. As the reaction temperature increases, the degradation rate of phenol significantly accelerates [48]. When the reaction temperature reaches 50 °C, the degradation rate reaches its maximum, and phenol is almost completely removed within 90 min, with the degradation effects at 40 °C and 50 °C being essentially the same. These results demonstrate that the SBC-700-20%/PDS system exhibits excellent phenol degradation performance under both low and high temperature conditions, highlighting its broad adaptability in practical applications.

Figure 5b illustrates the efficiency trends of phenol degradation catalyzed by SBC-700-20% under different pH conditions. As shown in the figure, the degradation rate and efficiency of phenol reach their maximum in a neutral environment (pH = 7), slightly decrease in an acidic environment (pH < 7), and significantly decline in an alkaline environment (pH > 7) [49]. The decreasing degradation rate resulting from low pH was associated with the inhibiting generation of superoxide radicals as well as hydroxyl radicals excited from the surface of the sample. While, when the pH was above 7, hydroxyl radical derived from the activated persulfate was easily annihilated by the negative ions. Furthermore, the negative ions dissociated from phenol also underwent strong electrostatic repulsion from the surface of biomass carbon. This resulted in a decreasing phenol degradation. Nevertheless, the final degradation rate remains above 87%. These results demonstrate that the SBC-700-20%/PDS system exhibits excellent catalytic degradation capability for phenol across a wide pH range, highlighting its broad applicability in practical applications.

To investigate the effect of PDS dosage on phenol degradation efficiency, experiments were conducted by adding different concentrations of PDS (0.5, 1, 1.5, and 2 mmol) to phenol solutions under the action of the same mass of SBC-700-20% catalyst, and the results are shown in Figure 5c. The experimental data indicate that when the PDS dosage was increased from 0.5 mmol to 1 mmol, the phenol removal rate significantly improved, with the final degradation rate rising from 93% to 98%. However, when the PDS dosage was further increased to 1.5 mmol and 2 mmol, the phenol degradation rate noticeably decreased. This phenomenon can be attributed to the excessive persulfate concentration, which enhanced the quenching effect of residual persulfate on free radicals, thereby inhibiting the degradation reaction.

In addition, the effect of SBC dosage (0.25, 0.5, 0.75, and 1 g/L) on the catalytic degradation of phenol was investigated, and the results are shown in Figure 5d. As the SBC dosage increased, the degradation rate significantly improved. When the dosage was increased from 0.25 g/L to 0.5 g/L, the catalytic degradation rate of phenol reached 89% within 15 min. Further increasing the dosage to 0.75 g/L and 1 g/L resulted in the complete disappearance of phenol after 30 min of treatment, with the degradation rate at 1 g/L being higher than that at 0.75 g/L. Taking into account factors such as degradation efficiency, reaction performance, and cost, the optimal dosage of SBC-700-20% was determined to be 0.5 g/L. It can be inferred that as the biochar dosage increased, phenol and PDS were sufficiently adsorbed in a short time, thereby accelerating the reaction process.

### 3.3. Circulation Experiment

**Figure 6:**
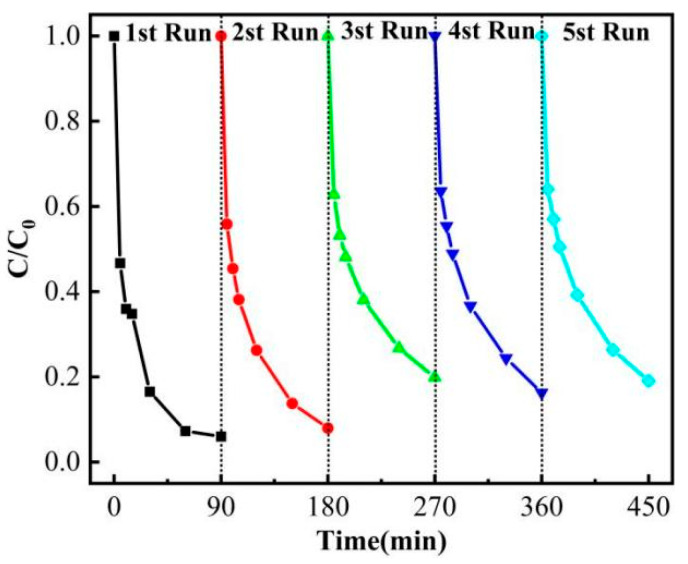
Cyclic experiment results.

In practical applications, the stability and recyclability of catalysts have always been the core focus of research. Figure 6 presents the cyclic performance test results of SBC-700-20% under the same experimental conditions. After each degradation experiment, SBC-700-20% was filtered and separated from the solution, washed, dried at 80 °C using a blast dryer, and regenerated in a tube furnace at 300 °C for 1 h, before being used in the next cycle of experiments. The cyclic test results indicate that after four cycles, the degradation rate of phenol by SBC-700-20% decreased from the initial 97.1% to 81%. Although the degradation rate and efficiency showed some decline, SBC-700-20% still demonstrated a relatively high catalytic degradation effect on phenol and good cyclic stability, suggesting its potential for long-term performance in practical applications.

### 3.4. Degradation Mechanism Analysis

#### 3.4.1. Radical Trapping Experiments and Electron Spin Resonance (ESR) Analysis

**Figure 7:**
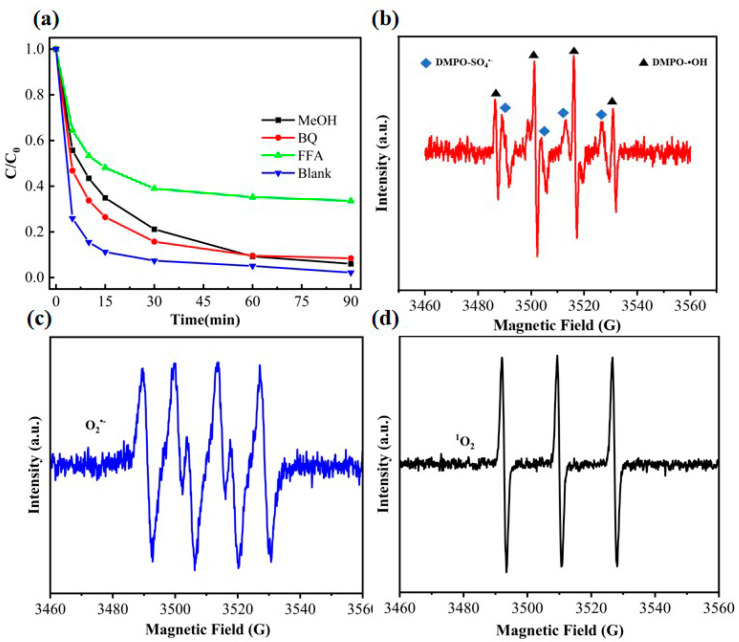
(**a**) Results of radical scavenging experiments; (**b**–**d**) ESR spectra.

To investigate the role of different active substances in the SBC/PDS degradation system, this study utilized various radical scavengers [50] for capture experiments, with the results depicted in Figure 7a. The experimental results indicate that the addition of methanol significantly inhibited the degradation rate of phenol, yet the final degradation rate still reached 94%. This suggests that SO_4_^•−^ and •OH play a certain role in promoting the catalytic degradation of phenol, but they are not the primary active species. Compared to methanol, the inhibitory effect of p-benzoquinone on the reaction rate was relatively weaker, with the final degradation rate slightly decreasing to 91.6%. These two sets of data collectively indicate that the SBC-700-20%/PDS system may not rely on the radical pathway as the main degradation mechanism. Notably, the inhibitory effect of furfuryl alcohol (FFA) on the reaction was the most significant, leading to a sharp decline in the degradation rate and efficiency of phenol. After 90 min of treatment, the phenol degradation rate was only 67.3%, further confirming that singlet oxygen (^1^O_2_) is the primary active substance in the degradation process. Analysis of the active substances reveals that singlet oxygen plays a dominant role in the degradation process, and the degradation mechanism primarily depends on non-radical pathways (^1^O_2_ and electron transfer), which is highly consistent with previous research findings [51,52]. Additionally, the introduction of sulfur effectively breaks the electron transport inertia of biochar, significantly enhancing the electron exchange capability of the catalyst, thereby driving the reaction efficiently along the non-radical pathway.

To further investigate the generation mechanism of active species in the catalytic reaction, this study employed electron spin resonance (ESR) spectroscopy, using DMPO and TEMP as trapping agents, and the results are shown in Figure 7b–d. The ESR test results reveal significant signals for DMPO-SO_4_^•−^ and DMPO-•OH, confirming the simultaneous generation of sulfate radical (SO_4_^•−^) and hydroxyl radical (•OH) in the SBC/PDS system. Additionally, DMPO successfully trapped superoxide radical (O_2_^•−^), displaying a characteristic quartet ESR signal. Meanwhile, TEMP, as a specific trapping agent for singlet oxygen (^1^O_2_), detected a strong ^1^O_2_ signal in the tests, and its classic triplet spectrum (1:1:1) was observed, further verifying the continuous generation of ^1^O_2_ during the reaction.

The ESR analysis results indicate that both free radical and non-free radical pathways coexist in the process of phenol degradation in this system. Based on the ESR test results and related literature, this study deduces the generation mechanism of active species as follows: the SO_4_^•−^ generated during activation reacts with water to produce •OH, while dissolved oxygen in the solution gains electrons to form O_2_^•−^, which further transforms into ^1^O_2_ through self-degradation. These four active species (SO_4_^•−^, •OH, O_2_^•−^, and ^1^O_2_) work synergistically to promote the efficient mineralization of pollutants. The results of the ESR and trapping experiments are highly consistent, confirming that the catalytic degradation of phenol by biochar in the PDS system involves both free radical and non-free radical pathways, with the non-free radical pathway being the dominant mechanism.

#### 3.4.2. Electrochemical Impedance Spectroscopy (EIS) and Density Functional Theory (DFT) Analyses

**Figure 8:**
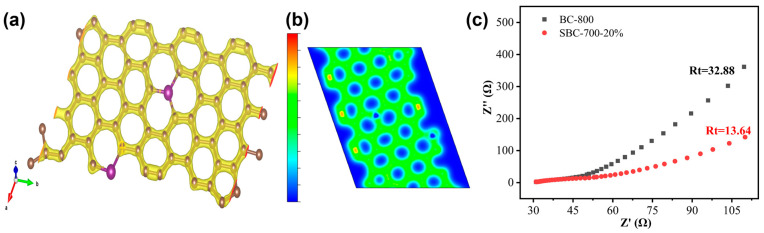
Differential charge density maps of the SBC: (**a**) 3D and (**b**) 2D; (**c**) impedance analysis.

To validate the impedance changes of the catalyst before and after doping, the differential charge density diagram of SBC-700 was obtained using DFT, as illustrated in Figure 8a,b. In SBC, sulfur primarily exists in the form of thiophene sulfur, which modulates the charge density distribution of adjacent carbon atoms, thereby breaking the catalytic inertia of biochar. This facilitates accelerated electron circulation and transfer, as well as enhances conductivity. Furthermore, to corroborate the theoretical calculations derived from DFT and to provide supplementary analysis of the surface resistance changes before and after biochar doping, an open circuit potential model was employed to conduct surface electrochemical impedance spectroscopy (EIS). The ZView model was utilized to construct the equivalent circuit diagram. The charge transfer resistance (Rt) and Nyquist radius of the biochar were simulated and analyzed, with the results depicted in Figure 8c. The Rt decreased steadily from 32.88 to 13.64 before and after doping. The introduction of sulfur reduced the impedance of the biochar and increased the rate of electron transfer, thereby promoting the reaction along a non-radical pathway. The EIS test results are thus consistent with the DFT calculations.

**Figure 9:**
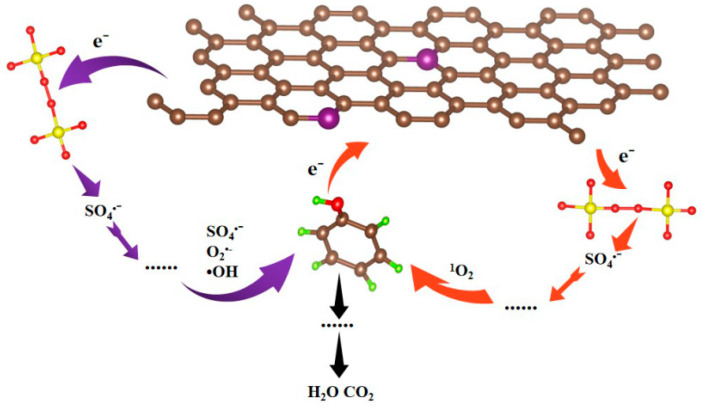
Proposed degradation mechanism of phenol in the SBC/PDS system.

Based on the characterization and DFT results, the mechanism of catalytic phenol degradation by the SBC/PDS system was proposed, as illustrated in Figure 9. The thiophene sulfur structure in the SBC enhances the catalytic activity and conductivity of the biochar by modulating the charge distribution of adjacent carbon atoms. Initially, phenol and PDS are simultaneously adsorbed onto the biochar. As an electron-rich organic compound, the electrons in the hydroxyl group of phenol are captured by PDS through the biochar. Upon acquiring these electrons, the O-O bond in PDS breaks, generating sulfate radicals (SO_4_^•−^). These radicals subsequently undergo a series of reactions to produce hydroxyl radicals (•OH) and superoxide radicals (O_2_^•−^), which participate in the free radical pathway degradation. Additionally, the formation of singlet oxygen (^1^O_2_) is involved in the non-radical pathway degradation. These four active species further attack the electron-deficient phenol, breaking it down into small molecular organic compounds, which are eventually oxidized into carbon dioxide (CO_2_) and water (H_2_O). This dual-pathway mechanism highlights the efficiency of the SBC/PDS system in phenol degradation.

## 4. Conclusions

Sulfur-doped biochar (SBC) was successfully synthesized through co-pyrolysis under varying conditions, and its efficacy in phenol degradation using activated persulfate was systematically investigated under different parameters. The results demonstrate that the optimal preparation conditions for SBC were achieved at a pyrolysis temperature of 700 °C for 2 h with a sulfur doping ratio of 20%. Under these conditions, the synthesized biochar exhibited exceptional catalytic performance, achieving a phenol degradation efficiency of 97% within 90 min. Characterization analyses revealed that the prepared biochars consistently exhibited lamellar structures. While the specific surface area of the sulfur-doped biochars showed a modest increase, the degree of structural defects was significantly enhanced, which proved to be more favorable for catalytic phenol degradation. EIS tests and DFT calculations further indicated that sulfur doping altered the electron density distribution of the biochar and reduced its surface electrochemical impedance, facilitating rapid electron transfer and enhancing catalytic activity. Additionally, the SBC demonstrated excellent cyclic catalytic performance, highlighting its potential for practical applications and reducing economic costs associated with catalyst regeneration. Based on these findings, a plausible degradation mechanism for phenol in the SBC/persulfate system was proposed, providing valuable insights into the underlying catalytic processes. This study underscores the potential of sulfur-doped biochar as an efficient and sustainable catalyst for environmental remediation applications.

## Figures and Tables

**Table 1:** Structural properties of different materials.

Sample	Specific Surface Area (m^2^/g)	Mesopore Volume (cm^3^/g)	Average Pore Size (nm)
SBC-500	711	0.25	1.79
SBC-600	718	0.26	1.74
SBC-700	756	0.16	1.86
SBC-800	730	0.33	2.91
BC-800	706	0.30	3.10

**Table 2:** Elemental composition of SBC-700 and BC-800.

Element.	SBC-700	BC-800
C (at%)	88.15	86.37
O (at%)	5.2	9.83
S (at%)	3.15	1.04
N (at%)	2.25	1.98

## Data Availability

Data are contained within the article and Supplementary Materials.

## Supplementary Materials

The following supporting information can be downloaded at: https://www.mdpi.com/article/10.3390/nano15130979/s1, Table S1. The relative content of major elements in the doped biochar.
