# Supplementary material for: Enhanced Degradation of Phenol in Aqueous Solution via Persulfate Activation by Sulfur-Doped Biochar: Insights into Catalytic Mechanisms and Structural Properties
Source: Nanomaterials (Basel). 2025 Jun 24;15(13):979. doi: 10.3390/nano15130979 (PMC12250925; doi:10.3390/nano15130979)
Supplement: Supplementary file 1 [file nanomaterials-15-00979-s001.zip › nanomaterials-3687089-supplementary.pdf]

Enhanced Degradation of Phenol in Aqueous Solution via Persulfate Activation by Sulfur-Doped Biochar: Insights into Catalytic Mechanisms and Structural Properties

Guanyu Wang [1], Lihong Kou [1], Chenghao Li [2], Bing Xu [2,*] and Yuanfeng Wu [2,*]

1. China Coal Science and Technology Research Institute Co., Ltd., Beijing 100013, China

2. Henan Key Laboratory of Coal Green Conversion, College of Chemistry and Chemical Engineering, Henan Polytechnic University, Jiaozuo 454003, China

* Correspondence: xubinghpu@163.com (B.X.); wuyuanfeng2015@gmail.com (Y.W.)

2.1 Reagents and Instruments

Reagents: Sulfur powder, potassium persulfate (PDS), phenol, hydrochloric acid, and other reagents were of analytical grade, and all solutions used in the experiments were prepared with deionized water.

Instruments: The following instruments were employed in this study: a shaker (Model: HZ 9211KB, Changzhou Yineng Experimental Instrument Co., Ltd.), a muffle furnace (Model: LX1314, Tianjin Lebo Terry Instruments Co., Ltd.), an electrochemical workstation (Model: CHI760, Shanghai Chenhua Instrument Co., Ltd.), an ultraviolet-visible (UV-Vis) spectrophotometer (Model: UV-3600Plus, Shimadzu Corporation), a confocal Raman microscopy system (Model: inVia, Renishaw plc), a scanning electron microscope (SEM, Model: Merlin Compact, Carl Zeiss NTS GmbH), and an X-ray photoelectron spectrometer (XPS, Model: ESCALAB 250Xi, Thermo Fisher Scientific Inc.).

2.2 Preparation and Modification of Biochar

2.2.1 Preparation of BC

The peanut shells were thoroughly washed, dried in an oven at 80℃, ground into powder, and sieved through a 60-mesh sieve. The sieved peanut shell powder was placed in a crucible and heated in a muffle furnace to the target temperature at a heating rate of 5℃/min, maintaining the temperature for 4 hours. After naturally cooling to room temperature, the resulting biochar was soaked in dilute hydrochloric acid for 12 hours to remove ash, followed by repeated washing with deionized water until the washing water reached neutrality. The biochar was then filtered and dried to obtain the final product. The biochar samples were named based on their carbonization temperature. For instance, BC-800 denotes biochar carbonized at 800℃.

2.2.2 Preparation of Sulfur-Doped Biochar (SBC)

A specific mass of sulfur powder (0.1g, 0.2g, 0.3g, 0.4g) and 1g of biochar were separately weighed, thoroughly ground in a mortar, and transferred to a crucible. The crucible was placed in a muffle furnace and heated to predetermined temperatures (500℃, 600℃, 700℃, 800℃) for specified durations (1h, 2h, 3h, 4h), followed by cooling to room temperature. The modified biochar samples were named as follows: based on carbonization temperature, they were labeled SBC-500, SBC-600, SBC-700, and SBC-800; based on carbonization time, the biochar samples prepared at 700℃ were named SBC-700-1h, SBC-700-2h, SBC-700-3h, and SBC-700-4h; based on the mass ratio of sulfur powder to biochar, the modified biochar samples carbonized at 700℃ were named SBC-700-10%, SBC-700-20%, SBC-700-30%, and SBC-700-40%, respectively.

2.3 Experimental Method

2.3.1 Catalytic Degradation Experiment

A 100 mL phenol solution with a concentration of 50 mg/L was added to a 500 mL conical flask. Subsequently, 0.5 g/L of BC or SBC samples and 1 mM PDS were simultaneously introduced into the solution. The degradation experiment was conducted in a shaker maintained at a constant temperature of 30°C. At specified intervals, 4.5 mL of the phenol solution was sampled and filtered through a 0.45 μm organic filter membrane. The absorbance of the filtrate was measured at a wavelength of 270 nm using a UV spectrophotometer. The phenol concentrations and removal rates were calculated based on the standard curve, which will be detailed in the following section. The effects of various parameters, including catalyst dosage, persulfate dosage, pH, temperature, doping ratio, and carbonization temperature, on the degradation performance were systematically investigated. The phenol detection method was as follows: phenol solutions with concentrations of 5, 10, 20, 30, 40, and 50 mg/L were prepared. The absorbance of these solutions was measured using an ultraviolet spectrophotometer (UV-3600Plus, Shimadzu, Japan), and a standard curve was plotted and fitted. The fitting equation, Formula (S1), is as follows, with a coefficient of determination R^2=0.9999:

$$Y=0.0165X+0.01418 \qquad (S1)$$

where Y represents the absorbance values, and X represents the concentration values of phenol.

2.3.2 Recycling Experiment

The recyclability of the biochar catalysts was evaluated. At the conclusion of the degradation experiment, the solution was filtered, and the collected catalyst was rinsed three times with deionized water. The catalyst was then placed in a muffle

furnace and maintained at a constant temperature of 350℃ for 60 minutes. After naturally cooling to room temperature, the catalyst was collected. For the recycling experiment, the mass of the catalyst was adjusted to 0.05 g to ensure consistency in subsequent degradation tests.

Table S1. The relative content of major elements in the doped biochar

Sample Name	Pyrolysis Temperature (°C)	Sulfur Doping Amount (wt%)	C%	O%	S%	N%
SBC-500-20%	500	20%	86.50	6.80	2.80	2.60
SBC-600-20%	600	20%	87.30	6.00	3.00	2.40
SBC-700-20%	700	20%	88.15	5.20	3.15	2.25
SBC-800-20%	800	20%	89.00	4.50	2.90	1.90
SBC-700-10%	700	10%	89.50	5.60	1.60	2.40
SBC-700-30%	700	30%	86.50	4.70	4.80	2.00
SBC-700-40%	700	40%	84.60	4.20	6.40	1.80
